# Providers of antenatal care services in Ghana: evidence from Ghana demographic and health surveys 1988–2014

**DOI:** 10.1186/s12913-017-2145-z

**Published:** 2017-03-14

**Authors:** Kwamena Sekyi Dickson, Eugene Kofuor Maafo Darteh, Akwasi Kumi - Kyereme

**Affiliations:** Department of Population and Health, University of Cape Coast, Cape Coast, Ghana

**Keywords:** Providers, Antenatal care services, Utilisation, Women, Ghana

## Abstract

**Background:**

Antenatal care is one of the three most essential care - antenatal, delivery and post-natal, given to women during pregnancy and has the potential to contribute towards the achievement of the Sustainable Development Goal (SDG) target 3.1- reducing the global maternal mortality ratio to less than 70 per 100,000 and target 3.8 – achieve universal health coverage. The main objective is to examine the contribution of the various providers of antenatal care services in Ghana from 1988 to 2014.

**Methods:**

The study uses data from all the six rounds of the Ghana Demographic and Health Survey (GDHS). Binary logistic regression models were applied to examine the association between background characteristics of respondents and providers of antenatal care services.

**Results:**

The results show that majority of antenatal care services were provided by nurses over the period under review. The proportion of women who received antenatal care services from nurses improved over the period from 55% in 1988 to 89.5% in 2014. Moreover, there was a decline in antenatal care services provided by traditional birth attendants and women who did not receive antenatal care services from any service provider over the years under review. It was observed that women from rural areas were more likely to utilise antenatal care services provided by traditional birth attendants, whilst those from urban areas were more likely to utilise antenatal care from doctors and nurses.

**Conclusion:**

To further improve access to and utilisation of antenatal care services provided by nurses and doctors it is recommended that the Ghana Health Service and the Ministry of Health should put in place systems aimed at improving on the quality of care given such as regular training workshops for health personnel and assessment of patient’s satisfaction with services provided. Also, they should encourage women in rural areas especially those from the savannah zone to utilise antenatal care services from skilled providers through social and behaviour change communication campaigns.

## Background

Maternal mortality is one of the greatest development and health concerns facing developing countries [1]. Approximately 800 women die every day from complications arising from pregnancy and childbirth [2]. However, the proportion of women dying due to complications during pregnancy and childbirth, including severe bleeding after childbirth globally reduced by almost 50% from an estimated 523,000 in 1990 to 210,000 in 2014. Almost all of these deaths (99%) occur in developing countries, and most can be avoided as the necessary medical interventions exist and are well known [2]. In Africa, dying from complications from pregnancy-related causes during a woman’s lifetime is 1 in 40 compared to 1 in 3300 in Europe and 1 in 190 globally [3]. Maternal mortality ratio in Ghana increased from 173 in 2011 [4] to 319 in 2015 [2]. With a ratio of 319 in 2015[], Ghana missed the opportunity to achieve MDG5. Evidence suggests that the antenatal period offers opportunities to reaching pregnant women with a number of interventions such as Antenatal Care (ANC) that may be vital to the health of the woman and her unborn baby [5–7] and ultimately reduce maternal mortality ratios [5, 7].

Antenatal Care (ANC) is one of the three most essential care given to women during pregnancy [2] and a key indicator of the Sustainable Development Goal (SDG) 3 target 3.1 - reducing the global maternal mortality ratio to less than 70 per 100,000. The main aim of the antenatal care is to prepare women for birth and motherhood as well as manage, check, identify and alleviate the three types of health problems during pregnancy that affect mothers and babies. They include complications of pregnancy itself, pre-existent conditions that worsen throughout the pregnancy period and the effects of unhealthy lifestyles [8]. Conventionally, antenatal care was recommended for developing countries along the path of those used in developed countries, with only slight amendments made to fit the local context because of its potential of helping to reduce maternal mortality and improving maternal and child health [9]. A standard of four antenatal visits is recommended for a healthy pregnant woman from a skilled health care provider [9]. A skilled attendant is defined by the WHO [9] as a qualified health professional who has been trained and educated with expertise to identify, provide and manage normal pregnancies and make referral of difficulties with pregnant women and newborns such as a doctor, midwife, or nurse. Skilled providers have also been explained to include doctors, nurse/midwives, and community health officer/nurses [10]. Skilled provider can identify complications and help to manage the situation. Despite the importance of antenatal care and all its potential in helping to reduce maternal mortality, antenatal care has been underused even when made available [11].

Globally, it was estimated in 2014 that six out of ten pregnant women made at least four ANC visits; nine out of ten in the Americans; seven out of ten in the South – East Asia; four out of ten in the Eastern Mediterranean and Africa [2]. In sub–Sahara Africa, pregnant women who make four or more antenatal care visits vary from 12% in Ethiopia [2]; 35 per cent in Rwanda, 47% in Kenya, 62% in Cameroon to 87% in Ghana [10]. Although antenatal coverage is high, the percentage reporting at least four visits is low. For instance, in Rwanda, while 98% of women reported at least one antenatal care visit, 35% of women reported four or more ANC visits [2]. Low utilisation of antenatal care services can affect the adequacy of information and services given to women reporting for care thus leading to poor maternal mortality outcomes [12]. For instance, evidence from sub–Saharan African countries shows that less than half of women who utilise antenatal care services were not informed about the danger signs of pregnancy complications. These percentages range from 10% in Rwanda, Mali 29%, Cameroon 38%, Uganda 35%, Zimbabwe 49% to 73% in Zambia [13]. In Ghana, two–thirds of women who utilise antenatal care received information about the danger signs of pregnancy complications [13].

In spite of the fact that the national coverage of antenatal care service for the recommended four or more ANC visits is above the global average of 64% in Ghana [2, 10], there still exists urban – rural differences and regional disparities among the providers of antenatal care services [14, 15] with women not getting antenatal care from skilled care providers [16, 17]. There are still some pregnant women who did not have ANC. Evidence shows that regardless of the socio – economic and demographic factors, women enrolled in the National Health Insurance Scheme (NHIS) make more antenatal visit compared with those who are not enrolled [18]. Women who are educated, living in urban centres and are wealthy are more likely to attend antenatal care visits than those uneducated, those from poorer households and those in rural areas [19]. Furthermore, [20] confirmed that the wealth status, educational level, age, transportation, health insurance, and the number of children the pregnant women already have, also influence the use of antenatal care services [20]. Abor, Abekah – Nkrumah, Sakyi, Adjasi and Abor [14] affirmed these findings when they were looked at the socio – economic determinants of maternal health care utilisation. The question one may ask is why are some pregnant women not utilising the services of any ANC service provider? Why do some pregnant women utilise antenatal care services from traditional birth attendants?

The available evidence suggests that there is paucity of evidence on the providers of antenatal care services in Ghana. This paper contributes to the discourse on antenatal care by examining the providers of antenatal care services in Ghana from 1988 to 2014.

## Methods

### Setting

The republic of Ghana is located on the West African Coast and has a total land area of 238, 533 square kilometres. Ghana is bordered by three francophone countries: Burkina Faso to the north, Togo on the east and Cote d’Ivoire on the west [15]. Ghana is a low-lying country except for a series of hills on the eastern border and Mountain Afadjato, the maximum point (883 metres) above sea level which is west of Volta Region. Ghana can be divided into three ecological zones namely; Savannah zone, Forest Zone and the Sandy Coastline supported by coastal plains (coastal zone). The main ethnic groups in Ghana are namely; Akan (47.5%), Mole-Dagbani (16.6%), Ewe (13.9%), Ga–Dangme (7.4%), Gurma (5.7%), Guan (3.7%), Grusi (2.5%), Other (1.4%) and Mande (1.1%) [21]. Ghana has about 51% of its population in urban areas and 49% in rural areas. There are 3217 functional health facilities out of which 4 are teaching hospitals. There are also 9 regional hospitals, 3 psychiatric hospitals, 11 polyclinics, 59 Christian Health Association of Ghana (CHAG) hospitals, 10 Islamic hospitals, 96 government hospitals, 156 private hospitals, and 22 quasi-government hospitals, 389 maternity homes and 379 Community – based Head Planning and Services (CHPS) compounds. Majority of these health facilities are found in the urban areas [4, 21].

### Source of data

The Ghana Demographic and Health Survey (GDHS) 1988, 1993, 1998, 2003, 2008, and 2014 used standard DHS model questionnaire developed by the Measure DHS programme [15, 22–26]. The Ghana Demographic and Health Survey is a nationwide survey which covers all ten regions and is conducted every five years. The survey is carried out by the Ghana Statistical Service and the Ghana Health Service with ICF International providing technical support for the survey through MEASURE DHS. The GDHS focuses on child and maternal health and is designed to provide adequate data to monitor the population and health situation in Ghana. The survey gathers data on various demographic and health issues including fertility, contraceptive use, child health, nutrition, malaria, HIV and AIDS, family planning, health insurance and maternal health; antenatal care, delivery care, and post-natal care. For the purpose of the study women with birth history were sampled. Thus, 4294 women in 2014, 2131 women in 2008, 2734 women in 2003, 2374 women in 1998, 1974 women in 1998 and 2701 women in 1988 [15, 22–26]. Permission to use the data set was granted by MEASURE DHS following the assessment of a concept note.

### Data analysis

The study uses providers of antenatal care (ANC) services as the dependent variable. The providers of the antenatal care services variable were derived from the response to the question “did you see anyone for antenatal care for this pregnancy? If YES: Whom did you see?” Responses were categorised as Doctor, Nurse, Nurse/Midwife, Auxiliary Midwife, Community Health Nurse/Officer, Traditional Birth Attendant (TBA), Traditional Health Volunteer, Village Health Volunteer, Other and No one. Respondents who chooses more than one provider, the provider with the highest rank is considered. For the purpose of the study, the providers of antenatal care were Doctor, Nurse (Nurse, Auxiliary Midwife, Nurse/Midwife were recoded as nurse) and Traditional Birth Attendant (Trained Traditional Birth Attendants, Traditional Birth Attendants were recoded as Traditional Birth Attendant) and No one. Village Health Volunteer, Traditional Health Volunteer and Community Health Nurse/ Officer were dropped because they were not found in all the six rounds of the survey.

The study made use of ten independent variables - maternal age, marital status, educational level, residence, wealth status, ethnicity, occupation, parity (Birth order), ecological zone and survey wave year. Maternal age was categorized into 7 age groups, thus, 15–19, 20–24, 25–29, 20–34, 35–39, 40–44, 45–49. Marital status was originally captured as Never married, Married, Living together, Widowed, Divorced and Not living together but was recoded as Single (Never married, Widowed, Divorced, Not living together), Married and Cohabitation (Living together). Educational level was classified into four categories: No education, Primary education, Secondary education and Higher education. Type of Residence coded as Urban or Rural. Wealth Quintile was categorised in Poorest, Poorer, Middle, Richer and Richest.

Ethnicity was recoded as Akan (Asante, Akwapim, Fante, and other Akan), Ga – Adangbe, Ewe, Northern Ethnic Groups (Guan, Mole – Dagbani, Grussi, Gruma, Hausa, Dagarti) and Other. Occupation was captured as Not working and Working. Parity (birth order) was captioned from a question that measured if respondents had ever given birth. Responses were categorised as Zero (prior to current pregnancy) One birth, Two births, Three births and Four births or more.

The ecological zone was originally coded as Western, Central, Greater Accra, Volta, Eastern, Ashanti, Brong – Ahafo, Northern, Upper East, and Upper West. Region of residence were re – coded to capture the general ecological zones as follows: Northern, Upper East, and Upper West regions were re - coded as the ‘Savannah zone’; the Brong – Ahafo, Ashanti and Eastern regions were designated as the ‘Forest zone’; while the Western, Central, Greater Accra and Volta regions were coded as the ‘Coastal zone’. Survey wave year was captured as 1988; 1993; 1998; 2003; 2008 and 2014.

The statistical software STATA version 13 was used to process the data. Some variables were recoded and renamed so that they would be consistent across all the rounds and all results were weighted. Univariate, bivariate and multivariate and line graphs were carried out. The dependent variable, the providers of antenatal care services, were coded 0 = No, and 1 = Yes. A discrete choice model was employed to show how the independent variables are related to the dependent variable. Precisely, the binary logistic regression was employed given that the model is the best fit for dichotomous variables and its ability to predict on a mixture of continuous and categorical variables. The binary logistic regression is based on the assumption that the dependent variable should be dichotomous in nature and the data should not have any outlier. For the descriptive statistics data was analysed in their individual survey wave years (e.g. 1988, 1993) but for the logistic regression the data from the individual survey wave years were merged and appended as a panel data set.

## Results

### Background characteristics of respondents

The mean age of the respondents was 28.6 years in 1988, and about 30 years in 2014 (see Table 1). The highest proportion of the respondents was from the coastal zone in all the years with the exception of 2003 and 2008, where most of the respondents were predominantly from the forest zone. About 72% of the respondents in 1988 and about 54% in 2014 were from rural areas. The results show that from 1988 to 1993, majority of the respondents had primary education whereas from 1998 to 2014, the highest proportions of respondents had secondary education. The distribution wealth status did not vary much over the years. It ranges from about 15% of respondents within the richest wealth status in 2008 to about 25% of respondents within the poorest wealth status in 2003 (see Table 1). More than 8 in 10 of the respondents in 1988 were married whilst about 61% reported being married in 2014. About 49% of the respondents had four births or more in 1988; this reduced to 39% of the respondents in 2014. The highest proportion of the respondents was from the Akan ethnic group (see Table 1).

**Table 1:** Background characteristics

	Years					
Variables	1988	1993	1998	2003	2008	2014
	*n* = 2701	*n* = 1974	*n* = 2374	*n* = 2743	*n* = 2131	*n* = 4294
	(%)	(%)	(%)	(%)	(%)	(%)
Age						
15–19	6.0	7.4	4.6	4.5	4.8	4.5
20–24	22.1	22.9	22.3	19.3	19.2	17.0
25–29	26.5	25.8	26.1	24.6	26.5	24.2
30–34	19.5	22.5	18.5	21.9	20.5	23.4
35–39	14.7	12.8	15.9	16.5	17.2	18.8
40–44	7.3	6.6	9.3	8.8	8.2	9.2
45–49	3.9	2.0	3.3	4.4	3.6	2.9
Ecological Zone						
Coastal Zone	45.6	39.0	45.9	37.1	39.7	45.2
Forest Zone	40.6	38.5	38.0	40.2	38.3	36.3
Savannah zone	13.8	22.5	16.1	22.7	22.0	18.5
Residence						
Urban	28.4	28.5	26.1	35.9	40.2	46.2
Rural	71.6	71.5	73.9	64.1	59.8	53.8
Education						
No education	44.1	40.2	36.5	38.7	30.6	26.1
Primary	50.7	54.1	20.3	22.3	24.4	19.6
Secondary	4.6	5.0	42.1	37.9	42.6	49.7
Higher	0.6	0.7	1.1	1.1	2.4	4.6
Wealth Status						
Poorest	-	17.6	13.5	24.5	22.8	21.0
Poorer	-	15.7	19.7	21.1	21.9	20.3
Middle	-	18.4	23.8	20.2	19.1	20.0
Richer	-	23.9	23.2	17.9	20.8	19.6
Richest	-	24.4	19.8	16.3	15.4	19.1
Marital Status						
Single	11.8	8.8	12.3	10.9	12.5	16.8
Married	82.2	76.3	71.6	79.4	68.0	61.7
Cohabitation	6.0	14.9	16.1	9.7	19.5	21.5
Parity						
One birth	19.0	20.1	22.3	21.6	22.2	22.8
Two births	17.2	20.1	20.3	19.3	20.9	20.4
Three births	15.3	16.2	14.4	16.1	16.7	17.8
Four or more births	48.5	43.6	43.0	43.0	40.2	39.0
Ethnicity						
Akan	51.4	48.4	52.4	47.1	46.6	47.4
Ga/Dangme	8.3	6.8	7.6	7.6	5.0	6.4
Ewe	15.3	13.6	14.3	11.9	13.0	13.2
Northern Ethnic Group	15.3	28.7	23.1	26.4	31.7	31.1
Other	9.7	2.5	2.6	7.0	3.7	1.9
Occupation						
Not working^a^	45.6	20.0	13.8	11.3	10.4	17.5
Working	54.4	80.0	86.2	88.7	89.6	82.5
Total	100	100	100	100	100	100

Source: Computed from GDHS 1988, 1993, 1998, 2003, 2008 and 20

Not working ^a^ = not involved in paid employment

### Dynamics of antenatal care providers from 1988–2014

**Fig. 1:**
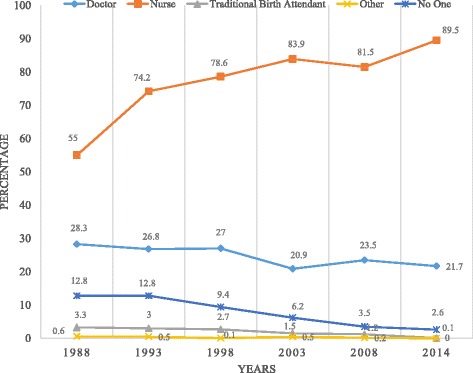
Trends in Providers of Antenatal Care from 1988–2014. Legend: Doctor, Nurse, Traditional Birth Attendants, Other and No one

Figure 1 shows that the proportion of antenatal care services provided by nurses increased from 55% in 1988 to about 90% in 2014. The percentage of women who received antenatal care services from a traditional birth attendant reduced from 3% in 1988 to 0.1% in 2014 whilst the percentage of women who did not receive antenatal care reduced from 13% in 1988 to 3% in 2014.

### Binary logistic regression

It is observed that likelihood of women utilising antenatal care services from a doctor varied by age. For instance, women aged 35–39 were more likely to utilise the service of a doctor than women aged 20–24 (OR = 1.54; *p* < 0.001). Also, women from the savannah zone were less likely to use the services of a doctor during antenatal care services compared to those from the coastal zone (OR = 0.47, *p* < 0.001) (see Table 2). Women with higher education (OR = 2.40, *p* < 0.001), married (OR = 1.20, *p* < 0.05), with richest wealth status (OR = 1.88, *p* < 0.001) and from Ga/Dangme ethnic group (OR = 1.26, *p* < 0.05) were more likely to utilise the services of a doctor during antenatal care services than women with no education, single, poorest wealth status and from the Akan ethnic group (see Table 2). For residence, the results show that women from rural areas were less likely to use the services of a doctor during antenatal care compared to those from urban areas (OR = 0.59, *p* < 0.001). Also, women with four births or more were less likely to utilise the services of a doctor during antenatal care than the reference (one birth) (OR = 0.60, *p* < 0.001). It was observed that utilisation of antenatal care services from doctors differed by survey wave year. For instance, women from survey wave years 1998 (OR = 0.84, *p* < 0.10); 2003 (OR = 0.55, *p* < 0.000); 2008 (OR = 0.70, *p* < 0.000) and 2014 (OR = 0.53, *p* < 0.000) were less likely to utilise antenatal care services from a doctor compared to women from survey wave year 1988 (see Table 2).

**Table 2:** Logistic regression on providers of antenatal care services

Variable	Doctors	Nurses	TBA	No one
	Odds ratio (confidence interval)	Odds ratio (confidence interval)	Odds ratio (confidence interval)	Odds ratio (confidence interval)
*Age*				
15–19	0.90 (0.72–1.14)	0.83 (0.65–1.05)	0.63 (0.28–1.43)	1.19 (0.82–1.76)
20–24	Ref	Ref	Ref	Ref
25–29	1.18** (1.03–1.36)	1.01 (0.87–1.18)	0.93 (0.61–1.45)	0.73** (0.58–0.93)
30–34	1.49*** (1.26–1.75)	1.01 (0.85–1.21)	0.76 (0.43–1.32)	0.61** (0.46–0.81)
35–39	1.54*** (1.29–1.87)	1.08 (0.89–1.32)	1.02 (0.56–1.84)	0.51*** (0.37–0.69)
40–44	1.45** (1.15–1.81)	0.93 (0.74–1.16)	1.39 (0.73–2.63)	0.65** (0.47–0.91)
45–49	1.41** (1.03–1.93)	0.93 (0.70–1.23)	1.63 (0.77–3.43)	0.64** (0.43–0.96)
*Ecological Zone*				
Coastal zone	Ref	Ref	Ref	Ref
Forest zone	0.97 (0.88–1.08)	1.37*** (1.22–1.54)	1.71** (1.18–2.49)	0.66*** (0.54–0.82)
Savannah zone	0.47*** (0.39–0.561)	1.22** (1.03–1.45)	2.68** (1.51–4.74)	1.14 (0.88–1.47)
*Ethnicity*				
Akan	Ref	Ref	Ref	Ref
Ga/Dangme	1.26** (1.06–1.50)	0.65*** (0.54–0.78)	2.10** (1.28–3.46)	0.92 (0.62–1.36)
Ewe	1.01 (0.88–1.15)	0.92 (0.79–1.08)	1.01 (0.61–1.69)	1.51** (1.18–1.94)
Northern ethnic group	0.89 (0.76–1.03)	0.97 (0.83–1.15)	0.71 (0.42–1.21)	1.13 (0.88–1.46)
Other	0.82 (0.62–1.07)	0.88 (0.68–1.13)	0.80 (0.35–1.83)	1.52 ** (1.06–2.17)
*Education*				
No education	Ref	Ref	Ref	Ref
Primary	1.48*** (1.30–1.68)	1.5*** (1.33–1.72)	1.12 (0.78–3.46)	0.45*** (0.37–0.54)
Secondary	1.55*** (1.30–1.68)	2.00*** (1.73–2.27)	0.51** (0.33–0.79)	0.15*** (0.11–0.20)
Higher	2.40*** (1.82–3.16)	1.41** (1.02–1.96)	1	1
*Occupation*				
Not working	Ref	Ref	Ref	Ref
Working	1.10 (0.96–1.24)	1.09 (0.96–1.25)	1.18 (0.78–1.80)	0.78** (0.64–0.96)
*Marital status*				
Single	Ref	Ref	Ref	Ref
Married	1.20** (1.04–1.38)	1.04 (0.89–1.22)	1.47 (0.83–2.60)	0.56*** (0.44–0.70)
Cohabitation	0.87 (0.74–1.03)	1.15 (0.96–1.39)	2.40** (1.31–4.43)	0.73** (0.55–0.97)
*Wealth status*				
Poorest	Ref	Ref	Ref	Ref
Poorer	1.09 (0.94–1.28)	0.94 (0.82–1.22)	1.08 (0.70–1.68)	1.36** (1.11–1.66)
Middle	1.26** (1.09–1.28)	0.88* (0.76–1.02)	1.65** (1.06–2.55)	1.33** (1.06–1.67)
Richer	1.31** (1.12–1.53)	0.78** (0.67–0.91)	2.49*** (1.64–3.76)	1.85*** (1.49–2.31)
Richest	1.88*** (1.60–2.21)	0.51*** (0.43–0.59)	2.59*** (1.65–4.05)	2.20*** (1.75–2.79)
*Residence*				
Urban	Ref	Ref	Ref	Ref
Rural	0.59*** (0.53–0.65)	0.79*** (0.71–0.89)	2.81*** (1.89–4.19)	3.51*** (1.59–3.43)
*Parity*				
One birth	Ref	Ref	Ref	Ref
Two births	0.89 (0.78–1.03)	0.80** (0.68–0.94)	1.02 (0.64–1.63)	1.57** (1.19–2.08)
Three births	0.82** (0.70–0.96)	0.86* (0.72–1.03)	0.67 (0.38–1.19)	1.77*** (1.30–2.42)
Four births or more	0.60*** (0.51–0.71)	0.82** (0.68–0.99)	0.89 (0.51–1.56)	2.41*** (1.75–3.31)
Survey wave year				
1988	Ref	Ref	Ref	Ref
1993	0.93 (0.81–1.05)	2.36*** (2.08–2.68)	0.02*** (0.02–0.03)	1.01 (0.84–1.19)
1998	0.84 ** (0.74–0.95)	2.74*** (2.43–3.10)	0.03*** (0.03–0.04)	0.84* (0.71–1.00)
2003	0.55*** (0.48–0.63)	4.21*** (3.71–4.78)	0.01*** (0.01–0.01)	0.57*** (0.47–0.68)
2008	0.70*** (0.62–0.80)	3.44*** (3.02–3.92)	0.01*** (0.01–0.02)	0.27*** (0.21–0.35)
2014	0.53*** (0.47–0.59)	8.05*** (7.08–9.15)	0.001*** (0.001–0.002)	0.21*** (0.17–0.26)

Source: computed from GDHS 1988, 1993, 1998, 2003, 2008 and 2014

*Ref* reference, *OR* Odds ratio *p < 0.10 **p < 0.05 ***p < 0.001

The findings suggest that women from the forest zone were more likely to use the services of a nurse during antenatal care services compared to those from the coastal zone (OR = 1.37, *p* < 0.001). For ethnicity, Ga/Dangmes were less likely to utilise the services of a nurse during antenatal care compared to Akans (OR = 0.65, *p* < 0.001). Women with secondary education (OR = 1.95, *p* < 0.001) were more likely to utilise the services of a nurse during antenatal care compared to women with no education (see Table 2). For residence, it was observed that women from rural areas were less likely to use the services of nurses during antenatal care as compared to those from urban areas (OR = 0.79, *p* < 0.001) (see Table 2). Utilisation of antenatal care services from nurses also differed by survey wave year with women from survey wave years 1993 (OR = 2.36, *p* < 0.000); 1998 (OR = 2.74, *p* < 0.000); 2003 (OR = 4.21, *p* < 0.000); 2008 (OR = 3.44, *p* < 0.000) and 2014 (OR = 8.05, *p* < 0.000) been more likely to utilise the services of a nurse during antenatal care compared to women from survey year 1988 (see Table 2).

Women within the richest wealth status (OR = 0.51, *p* < 0.001) were less likely to utilise the services of a nurse during antenatal care services compared to women within the poorest wealth status. Women with four births or more were also less likely to utilise the services of a nurse during antenatal care than the reference (one birth) (OR = 0.82, *p* < 0.05). It was observed that women from the savannah zone were more likely to utilise the service of a traditional birth attendant than women from the coastal zone (OR = 2.68, *p* < 0.05). Also, women from rural areas were more likely to use the services of a traditional birth attendant during antenatal care services compared to those from the urban areas (OR = 2.81, *p* < 0.0001) (see Table 2).

The likelihood of traditional birth attendants providing antenatal care services varied by education, marital status, and wealth status. For instance, women with secondary education were less likely to utilise the services of a traditional birth attendant compared to women with no education (OR = 0.51, *p* < 0.05) but cohabitating women (OR = 2.40, *p* < 0.05), with the richest wealth status (OR = 2.81, *p* < 0.001) were more likely to utilise the services of a traditional birth attendant during antenatal care services. Similar to utilisation of antenatal care services from nurses, there was a significant difference between survey wave year and utilisation of antenatal care services from traditional birth attendants. For instance, it was observed that women from survey wave years 1993 (OR = 0.02, *p* < 0.000); 1998 (OR = 0.03, *p* < 0.000); 2003 (OR = 0.01, *p* < 0.000); 2008 (OR = 0.01, *p* < 0.000) and 2014 (OR = 0.001, *p* < 0.000) were less likely to utilise theservices of a traditional birth attendant during antenatal care compared to women from survey year 1988 (see Table 2).

The likelihood of women not utilising antenatal care services varied by age. For instance, women aged 35–39 were less likely to utilise ANC services from any provider than women aged 20–24 (OR = 0.51, *p* < 0.001). Also, women from the forest zone were less likely to use ANC services compared to those from the coastal zone (OR = 0.66, *p* < 0.001) (see Table 2). Women with Secondary education (OR = 0.11, *p* < 0.001), married (OR = 0.56, *p* < 0.001), were less likely to utilise antenatal care services than women with no education, and single, (see Table 2). Again women with richest wealth status (OR = 2.20, *p* < 0.001) and from Ewe ethnic group (OR = 1.51, *p* < 0.05) were more likely not to utilise ANC services compared to women with poorest wealth status and from the Akan ethnic group. It was observed that women from rural areas were more likely not to utilise antenatal care services compared to those from the urban areas (OR = 3.51, *p* < 0.001). Further, women with four births or more were also more likely not to utilise antenatal care services during pregnancy than the reference (one birth) (OR = 2.41, *p* < 0.001) (see Table 2). The likelihood of women who did not receive antenatal care services varied by survey wave years. For instance, it was observed that women from survey wave years 1998 (OR = 0.84, *p* < 0.10); 2003 (OR = 0.57, *p* < 0.000); 2008 (OR = 0.27, *p* < 0.000) and 2014 (OR = 0.21, *p* < 0.000) were less likely to utilises antenatal care services compared to women from survey year 1988 (see Table 2).

## Discussion

The paper sought to examine the providers of antenatal care services in Ghana. Results of the study show that majority of women over the years received antenatal care services from nurses. The proportion of antenatal care services provided by nurses increased from 55% in 1988 to about 90% in 2014. Similar results were found by the work of [13] who found that women from Mozambique who utilised ANC services from nurses increased between 1997 and 2003.

The percentage of women who received antenatal care services from a traditional birth attendant reduced from 3% in 1988 to 0.1% in 2014 and the percentage of women who did not receive antenatal care from any ANC provider reduced from 13% in 1988 to 3% in 2014. This is consistent with the findings of [13]. This could possibly be due to the introduction of the Community–Based Health Planning Services (CHPS) compound. The CHPS compound system has improved access to health care, especially the hard to reach communities [4].

The results suggest that women from the forest zone were more likely to use the services of a nurse during antenatal care services compared to those from the coastal zone (OR = 1.37, *p* < 0.001). The likelihood of nurses providing antenatal care services varied by ethnicity. For example, Ga/Dangmes were less likely to utilise the services of a nurse during antenatal care compared to Akans (OR = 0.65, *p* < 0.001). This confirms the studies [27, 28] which observed that women from major ethnic groups were more likely to receive antenatal care services from skilled providers.

With residence, it was observed that women from rural areas were less likely to use the services of doctors (OR = 0.79, *p* < 0.001) and nurses (OR = 0.59, *p* < 0.001) during antenatal care as compared to those from the urban areas. This is in line with [13, 29]. The findings also show that women from rural areas were more likely not to utilise antenatal care services from any provider compared to those from the urban areas (OR = 3.51, *p* < 0.001). Again, women with four births or more were also more likely not to utilise antenatal care services from any provider during pregnancy than the reference (one birth) (OR = 2.41, *p* < 0.001).

The likelihood of traditional birth attendants providing antenatal care services varied by education, marital status, and wealth status. For instance, women with secondary education were less likely to utilise the services of a traditional birth attendant compared to women with no education (OR = 0.51, *p* < 0.05). Similar results were found Le Meur, Gao, and Bayat [30]. Women with higher education may be exposed to the danger signs of pregnancy complications and know the merits of receiving ANC services from skilled personnel. Women with the richest wealth status (OR = 2.81, *p* < 0.001) were seen to be more likely to utilise the services of a traditional birth attendant during antenatal care services. This finding goes contrary to the findings of Ganle et al. [29] Wang, Alva, Wang, & Fort [13]. They found out women with richest wealth status were more likely to receive ANC services from skilled personnel and rather those from poorest wealth status utilising ANC services from traditional birth attendants.

The limitation of the data is that the Ghana Demographic and Health Survey uses a repeated cross-sectional design and the sample that were used were not carried to all the rounds, chances could be that different set of respondents were used for the survey in all the different rounds. Changes in the sample over time may have effects on the results due to inherent characteristics. It may be possible that women may visit more than providers but this is not covered.

## Conclusion

To further improve access to and utilisation of antenatal care services provided by nurses and doctors it is recommended that the Ghana Health Service and the Ministry of Health should put in place systems aimed at improving on the quality of care given such as regular training workshops for health personnel and assessment of patient’s satisfaction with services provided. Also, they should encourage women in rural areas especially those from the savannah zone to utilise antenatal care services from skilled providers through social and behaviour change communication campaigns. There is also the need to strengthen the skills of the staff working in the Community–Based Health Planning Services (CHPS) compounds to deliver quality ANC services to women especially those in rural areas.
